# Current perspectives of mitochondria-targeted antioxidants in cancer prevention and treatment

**DOI:** 10.3389/fcell.2023.1048177

**Published:** 2023-03-16

**Authors:** Roman A. Zinovkin, Konstantin G. Lyamzaev, Boris V. Chernyak

**Affiliations:** ^1^ Belozersky Institute of Physico-Chemical Biology, Lomonosov Moscow State University, Moscow, Russia; ^2^ The “Russian Clinical Research Center for Gerontology” of the Ministry of Healthcare of the Russian Federation, Pirogov Russian National Research Medical University, Moscow, Russia

**Keywords:** cancer development, oxidative stress, mitochondrial ROS, mitochondria-targeted antioxidants, cancer therapy

## Abstract

Oxidative stress nearly always accompanies all stages of cancer development. At the early stages, antioxidants may help to reduce reactive oxygen species (ROS) production and exhibit anticarcinogenic effects. In the later stages, ROS involvement becomes more complex. On the one hand, ROS are necessary for cancer progression and epithelial-mesenchymal transition. On the other hand, antioxidants may promote cancer cell survival and may increase metastatic frequency. The role of mitochondrial ROS in cancer development remains largely unknown. This paper reviews experimental data on the effects of both endogenous and exogenous antioxidants on cancerogenesis focusing on the development and application of mitochondria-targeted antioxidants. We also discuss the prospects for antioxidant cancer therapy, focusing on the use of mitochondria-targeted antioxidants.

## 1 Introduction

Pronounced oxidative stress based on elevated reactive oxygen species (ROS) production is typical for various cancer cells, especially for rapidly growing aggressive tumors (Ganesh et al., 2022). Excessive production of ROS in cancer cells depends on increased metabolic rate, oncogene-dependent rearrangement of the signaling pathways, and intermittent hypoxia in solid tumors. Stimulation of ROS production or inhibition of antioxidant defense is generally considered to be a promising antitumor strategy, and the prooxidant activity of many chemotherapeutic drugs is thought to contribute to their therapeutic effect (Trachootham et al., 2009). It has been shown that the selective antitumor effects of some well-known drugs, such as menadione and ascorbate (Bakalova et al., 2020) or phytochemicals as phenethyl isothiocyanate (Sehrawat et al., 2017), depend on their prooxidant activity. An important class of anticancer drugs was proposed by B. Kalyanaraman and colleagues, who conjugated various prooxidants with the cationic triphiphenylphosphonium residue to target mitochondria (Zielonka et al., 2017). Some of these compounds inhibit mitochondrial respiration and redox metabolism, which increases their prooxidant and antitumor activity (Kalyanaraman et al., 2018). The selective toxicity of mitochondria-targeted agents against cancer cells, at least in part, may depend on the induction of specific mitochondrial autophagy (mitophagy) (Kalyanaraman, 2020).

Cancer cells adapt to permanent oxidative stress by activating antioxidant enzymes but also probably become addicted to high levels of ROS (Kim et al., 2018). Addiction to oxidative stress refers to a more general phenomenon of the dependence of the survival of transformed cells on various stresses, including DNA damage stress, proteotoxic stress, metabolic stress, etc., as well as on the cellular stress response pathways. It has been named “non-oncogenic addiction” as opposed to the well-known oncogenic addiction, and both are important targets for anticancer therapy (Luo et al., 2009).

The addiction of some tumors to oxidative stress suggests that antioxidants may have some anti-cancer properties. There is a clear controversy between antioxidant therapy and the possible interference of antioxidants with chemotherapy and radiotherapy, which depend on the induction of oxidative stress (Ambrosone et al., 2020). Another problem with the use of antioxidants is related to their ability to inhibit anoikis (a specific form of programmed cell death caused by disruption of specific cell interactions with the extracellular matrix), which can lead to stimulation of metastasis. With these aspects in mind, in this review we will discuss the prospects for antioxidant cancer therapy, focusing on the use of mitochondria-targeted antioxidants.

**SCHEME 1:**
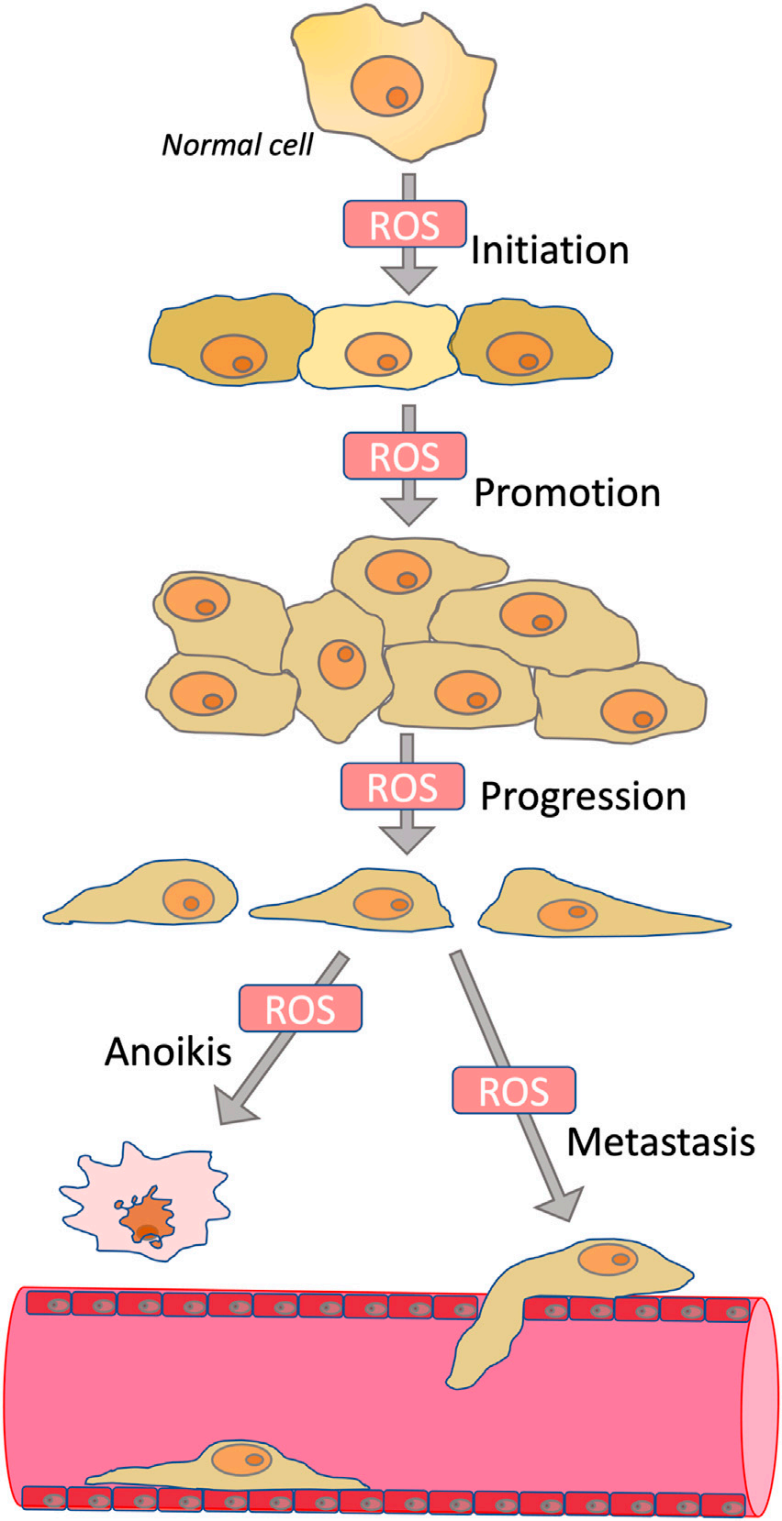
Role of ROS in carcinogenesis. ROS contribute to the initiation phase of carcinogenesis by stimulating mutagenesis; to the promotion phase by stimulating proliferation; to the progression phase by inducing epithelial-mesenchymal transition; and to the metastasis phase by stimulating invasion. At the same time, ROS are involved in anoikis, a mechanism of programmed cell death caused by detachment from the extracellular matrix. Because anoikis induction is an important mechanism for preventing metastasis, antioxidants may in some cases promote metastasis.

Tumor initiation, cell proliferation, migration, invasion, metastasis, angiogenesis, and drug resistance depend on ROS acting as mutagens, signaling transducers, or modulators of signaling pathways (Cheung and Vousden, 2022). Tumor initiation depends on mutations leading to activation of proto-oncogenes and inactivation of tumor suppressor genes, and these mutations are initiated by ROS-dependent oxidation of DNA (Schafer et al., 2009). Rapid cancer cell proliferation is depended on elevated ROS, primarily due to ROS-dependent inhibition of protein phosphatases (Lee et al., 2002; Salmeen et al., 2003), which leads to overstimulation of mitogenic signaling cascades mediated by kinases PI3K/AKT and MAPK (Kamata et al., 2005; Silva et al., 2011). ROS may also be responsible for the anchorage-independent growth of cancer cells (Weinberg et al., 2010) (Scheme 1). Angiogenesis, which is critical for the growth of solid tumors, is stimulated by ROS-dependent activation of hypoxia-induced factors (HIFs) (Chandel et al., 2000; Choudhry and Harris, 2018).

The early step in the stimulation of cancer cell motility and invasion leading to metastasis includes epithelial-mesenchymal transition (EMT), which is linked with the loss of cell-cell and cell-matrix contacts (Polyak and Weinberg, 2009; Ye and Weinberg, 2015). EMT begins with complex cytoskeletal rearrangement controlled by small Rho family GTPases (Ye and Weinberg, 2015). Later EMT is accompanied by increased expression of matrix metalloproteinases (MMPs), which cleave both cellular contact proteins and components of the extracellular matrix, thus stimulating intravasation of cancer cells followed by metastasis (Stallings-Mann and Radisky, 2007). TGF-β and NF-κB pathways, which are sensitive to ROS-dependent modulation, stimulate MMPs expression (Shimojo et al., 2013; Jiang et al., 2017; Hurst et al., 2022). Cancer stem cells (CSC) are a tiny subset of cancer cells that are uniquely capable of producing new tumors and are extremely resistant to many therapies. EMT is responsible for both the aggressiveness of tumors and their resistance to therapy (Mani et al., 2008; Shibue and Weinberg, 2017). EMT can contribute to the formation of CSC through modulation of intracellular signaling pathways and also by induction of autocrine signaling loops (Scheel et al., 2011). The tumor microenvironment (e.g., cancer-associated fibroblasts) makes a significant contribution to EMT activation and CSC formation (Diehn et al., 2009) [24]. Inflammation also stimulates EMT in tumors, while tumors, in turn, can promote inflammation and EMT in surrounding tissues (Giannoni et al., 2012). ROS are critical for signal transduction during EMT and inflammation as well as in the signal exchange between tumor and its microenvironment (Giannoni et al., 2012). Paradoxically, high resistance of CSC at least partially depends on a decreased level of ROS compared to mature tumor cells (Diehn et al., 2009).

Mitochondria are an important source of ROS (mtROS) in cancer as well as in non-transformed cells (Vyas et al., 2016). Most tumors have increased glycolysis to produce ATP even at aerobic conditions (the “Warburg effect”) (Warburg, 1925; Vaupel and Multhoff, 2021). Aerobic glycolysis, instead of more efficient but slower oxidative phosphorylation, is used by cancer cells to accelerate the production of nucleic acids, proteins, and lipids in support of rapid proliferation. In contrast to the view of Otto Warburg (Warburg, 1956), this effect is not necessarily caused by defects in mitochondrial respiration but rather by an oncogene-mediated rearrangement of cellular metabolic pathways. This rearrangement leads to increased uptake of glucose and activity of glycolytic enzymes, inhibition of pyruvate uptake into mitochondria, accumulation and excretion of lactate, which is accompanied by extracellular acidification. An important example of the relationship between oncogenes and mtROS is the KRAS oncogene, which is activated in colorectal cancer, lung cancer, and many others. Oncogenic mutations in protooncogene KRAS promote the Warburg effect, early steps of oncogenesis, and tumor growth. The KRAS-dependent increase in mtROS production was shown to be critical for cell proliferation, anchorage-independent growth, and growth of mouse lung adenocarcinoma *in vivo* (Weinberg et al., 2010). Liou et al. (2016) demonstrated that mutant KRAS-induced mtROS are responsible for the early formation of pancreatic precancerous lesions as well as tumor growth. It has been demonstrated that mtROS activate transcription factor NF-κB, resulting in the expression of epidermal growth factor receptor (EGFR) and its ligands (Liou et al., 2016). Mutations of important oncosuppressor p53 are frequently associated with the Warburg effect and increased mtROS levels, partially due to downregulation of TIGAR (TP53-induced glycolysis and apoptosis regulator) (Bensaad et al., 2006). Certain mutations in p53 have been shown to promote an aggressive transformed phenotype, presumably by stimulating respiration and mtROS (Lonetto et al., 2019).

Targeting excessive ROS production is a promising strategy for cancer treatment. In this review, we briefly discuss the effects of both endogenous and exogenous antioxidants on cancerogenesis, with a focus on the development and application of mitochondria-targeted antioxidants.

## 2 Antioxidants in cancer therapy

Living organisms exploit both enzymatic and non-enzymatic antioxidants for ROS scavenging. Main enzymatic systems include superoxide dismutase (SOD), catalase (CAT), glutathione peroxidase (GPx), and thioredoxin (Trx). Non-enzymatic antioxidants are represented by uric acid, vitamins C and E, coenzyme Q, glutathione (GSH), carotenes, and lipoic acid (the antioxidants are listed from highest to lowest concentration in human serum). Exogenous dietary antioxidants include multiple forms of carotenoids and flavonoids.

Oxidative stress significantly contributes to early steps in cancer initiation and progression. Thus the proper use of antioxidants is supposed to have a positive effect on cancer prevention (Liou and Storz, 2010). Moreover, cancer cells produce ROS that initiate survival signaling pathways, so antioxidant treatment may disrupt these pathways and promote cancer cell apoptosis. Antioxidants are also used in cancer therapy to prevent the excessive toxicity of chemotherapy drugs (Athreya and Xavier, 2017).

The use of Vitamin C in cancer treatment therapy is controversial (Nauman et al., 2018). The first efforts were made in the 1970s (Cameron and Pauling, 1974) and gave promising results for the treatment of various types of cancers with high doses of ascorbate applied per os and/or intravenously (IV). However, several limitations of this and similar studies have been shown. These trials were retrospective, there was no control group, no blinding, and the patients could inherently suffer from vitamin C deficiency (Padayatty and Levine, 2000). Placebo-controlled studies of oral vitamin C treatment reported no effect on cancer progression (Creagan et al., 1979). Thus, the role of Vitamin C in cancer treatment was dismissed for some time. It was later discovered that the IV route of administration results in high plasma concentrations of vitamin C that are unattainable through oral consumption. Since then, several clinical trials have demonstrated the benefits of IV vitamin C treatment for various cancers (Nauman et al., 2018). It is worth mentioning that the same antioxidant molecule may possess both antioxidant and pro-oxidant activities depending on its concentration and environmental conditions (Sotler et al., 2019). It is possible that high plasma ascorbate levels produce hydrogen peroxide, which stimulates pro-apoptotic pathways in cancer cells (Violet and Levine, 2017). Still, there is an urgent need for large-scale placebo-controlled randomized clinical trials to establish the potential of Vitamin C in cancer treatment.

Vitamin E is another antioxidant widely used in cancer research. In the long-term randomized controlled trial of 14,641 male physicians, daily uptake of vitamins C and E did not reduce the risk of prostate or any other cancer (Gaziano et al., 2009). Even more disappointing results were produced another large-scale randomized controlled Selenium and Vitamin E Cancer Prevention Trial (SELECT) enrolling 35,533 men. Selenium consumption exacerbated the risk of prostate cancer among men with high selenium status and had no effect on men with low selenium status. Vitamin E increased the risk of prostate cancer in men with low selenium (Kristal et al., 2014). Possibly these effects were due to selenium and Vitamin E overdose effects unrelated to their antioxidant effects, but the idea of chemoprophylaxis with dietary antioxidants was heavily compromised by this study. A possible explanation for the carcinogenic effect of Vitamin E was found in 2019 on rat models (Vivarelli et al., 2019). Vitamin E increased expression of phase-I activating cytochrome P450 (CYP) enzymes, which is associated with the generation of oxidative stress, causing DNA damage and increasing frequency of cell transformation caused by benzo [a] pyrene (Vivarelli et al., 2019).

N-acetylcysteine (NAC) is another antioxidant, the precursor of cysteine, which in turn is the rate-limiting metabolite in GSH synthesis. In pioneering work, NAC dietary supplementation of p53 knockout mice prevented lymphoma development and decelerated the growth of lung cancer xenografts (Sablina et al., 2005). These results were attributed to the antioxidant function of p53 protecting the genome from oxidation by ROS. Later findings confirmed the antitumorigenic activity of NAC in other mouse models but challenged the paradigm of the antioxidants preventing ROS-induced DNA damage and mutation (Gao et al., 2007). Instead, reduction of hypoxia-inducible factor (HIF-1a) by antioxidants has been shown to play a key role, and suppression of tumor angiogenesis has been proposed as the main effect of NAC (Gao et al., 2007).

Inflammation is always accompanied by sustained oxidative stress, resulting in oxidation of nucleotides (e.g., 8-oxoguanine formation), DNA damage, and a higher mutagenesis rate. NAC treatment in mice reduced 8-oxoguanine lesions and decreased the risk of developing colitis-associated colon cancer (Irrazabal et al., 2020). Tobacco smoke is an established carcinogenic factor, and also increases oxidative burden in the lungs of smokers. Oral NAC consumption (1.2 g daily, more than 6 months) improved some cancer-associated biomarkers, including 8-oxoguanine and lipophilic-DNA adducts in bronchoalveolar lavage cells (Van Schooten et al., 2002). Numerous studies support the use of NAC in the treatment of triple-negative breast cancer (Kwon, 2021). However, preclinical studies in mice demonstrated the carcinogenic effect of NAC, thus raising concerns regarding its clinical use (Sayin et al., 2014; Le Gal et al., 2015). In addition, NAC and vitamin E increased proliferation of tumor cells by decreasing ROS, DNA damage, and p53 expression in mouse and human lung tumor cells (Sayin et al., 2014). Inactivation of p53 increases tumor growth to a similar degree as antioxidants and abolishes the effect of antioxidants. Thus, antioxidants accelerate tumor growth by disrupting the ROS-p53 axis (Sayin et al., 2014). NAC also induced lung adenocarcinoma in aging mice (Breau et al., 2019). There is a possibility that NAC acts not only as an antioxidant but as a reducing agent, disrupting disulfide bonds in a cell, thus perturbing its homeostatic and signaling functions (Aldini et al., 2018).

The expression of the most important antioxidant enzymes (SOD, GPx, Trx, etc.) is controlled by transcription factor Nrf2 encoded by *NFE2L2* gene. Pharmacological agents can activate Nrf2 expression, and this could be a promising approach to decrease oxidative stress in cancer cells. Nrf2 is kept in the cytoplasm by its negative regulator, Keap1 protein, while pro-oxidants or xenobiotics destabilize Keap1-Nrf2 complex and the latter accumulates in the nucleus. Nuclear Nrf2 recognizes specific DNA sequences (antioxidant response elements, ARE) in gene promoters, thus stimulating their transcription (Motohashi and Yamamoto, 2004). The promoter of *NFE2L2* gene also contains ARE, thus creating a positive feedback loop (Kwak et al., 2002). Nrf2 induces expression of phase I and II drug metabolizing enzymes, and phase III drug transporters (Xu et al., 2005). Phase I enzymes convert a xenobiotic to a more polar molecule by oxidation, reduction, or hydrolysis. Phase II drug metabolizing enzymes conjugate resulting compounds to glycine or glutathione, or perform their methylation, sulphation, acetylation, or glucuronidation. Phase II enzymes include rate limiting enzymes in glutathione biosynthesis (GSH reductase, and GCL), thioredoxin reduction (Txn reductase), reduction of reactive intermediates (NAD(P)H: quinone oxidoreductase (NQO1) and epoxide hydrolase), and degradation of redox-active heme by heme oxygenase 1 (HO1) (Kaspar et al., 2009). Thus, phase II enzymes are often referred to as antioxidant enzymes because they maintain redox balance and thiol homeostasis (Dinkova-Kostova and Talalay, 2008). Nrf2 knockout mice have reduced glutathione levels and an increased oxidative burden (Beyer et al., 2008). These mice, compared to wild type control animals, are much more susceptible to carcinogenesis in stomach (Ramos-Gomez et al., 2001), bladder (Iida et al., 2007), and liver (Kitamura et al., 2007).

An important mechanism of the anticancer activity of Nrf2-dependent antioxidants is associated with the activation of protein tyrosine phosphatases (PTPs). These include, in particular, PTEN (phosphatase and tensin homologue deleted on chromosome 10), which is a tumor suppressor that is inactivated in many human cancers (Zhang et al., 2020). PTEN is a negative regulator of the PI3K/Akt signaling pathway that suppresses Ras- or ErbB-2-induced transformation. Various ROS, and mtROS in particular (Kim et al., 2018), inactivate PTEN by oxidizing critical cysteine residues. Peroxiredoxins (Prdxs), a family of small non-seleno peroxidases, as well as Trx, bind to PTEN and effectively protect it against oxidation, thereby inhibiting tumorigenesis (Cao et al., 2009; Zhang et al., 2020).

There are multiple examples of anticancer activities of Nrf2-inducing compounds. A natural organosulfur compound, sulforaphane, induces apoptosis in HT29 colon cancer cells *via* a p53 mechanism (Gamet-Payrastre et al., 2000), prevents UV-induced skin cancer (Dickinson et al., 2009; Alyoussef and Taha, 2019), prevents carcinogen-induced oral cancer (Bauman et al., 2016), and suppresses the growth of triple-negative breast cancer stem-like cells *in vitro* and *in vivo* (Castro et al., 2019). All the “Big Five” phytochemicals targeting cancer stem cells: curcumin, epigallocatechin-3-gallate (EGCG), sulforaphane, resveratrol, and genistein (Naujokat and McKee, 2021) are able to induce Nrf2 activation. According to the available recent clinical trials, these compounds demonstrated their therapeutic efficacy, alone or in combination with other cancer therapeutics, and in various types of cancer (Naujokat and McKee, 2021). Therefore, Nrf2 activation is favorable, at least for the inhibition of tumor initiation. However, prolonged Nrf2 activation in established cancer cells provides them benefits by enhancing antioxidant and detoxification capabilities, thus conferring therapeutic resistance to chemotherapy (Kitamura and Motohashi, 2018). In agreement with that, there are many examples of so-called Nrf2-addicted cancers bearing mutations in Keap1-Nrf2 system resulting in its permanent activation (reviewed in (Taguchi and Yamamoto, 2020)).

## 3 Antioxidants and metastasis

It is worth mentioning that solid cancer cells often enter the bloodstream but rarely produce active metastases. In a human melanoma xenograft model, cytoplasmic ROS levels were found to be higher in circulating melanoma cells and visceral metastatic nodules than in subcutaneous tumors, while mitochondrial ROS were higher in visceral metastatic nodules than in circulating cells and subcutaneous tumors (Piskounova et al., 2015). It has been suggested that these cells are subjected to oxidative stress, which limits their survival (Piskounova et al., 2015). It was shown that daily subcutaneous injections of NAC (200 mg/kg/day) promoted distant metastasis in this model. At the same time, NAC treatment did not enhance the growth of the subcutaneous tumors (Piskounova et al., 2015). A similar effect of NAC has been described in an endogenous mouse model of malignant melanoma (Le Gal et al., 2015). In this study, mice harboring the conditional oncogenic Braf allele and conditional knockout alleles for Pten were locally activated with 4-hydroxytamoxifen to initiate the development of melanoma. NAC administration doubled the number of lymph node metastases, only modestly increased lung metastases, and had no effect on the number or size of primary tumors. Later, the same research team demonstrated that NAC and vitamin E promote lung cancer metastasis in mice, in which Cre-adenovirus administration activates KRAS overexpression (Wiel et al., 2019). The frequency of lymph node metastasis was 6-7 times higher in antioxidant-treated mice compared to the control group, while stimulation of distant metastasis was less pronounced. The antioxidants did not affect mice survival or primary tumor growth, in agreement with the previous studies. In this study, as well as in the paper (Lignitto et al., 2019), the antioxidants stabilized the transcription factor BACH1, which is critical in the metastasis of lung cancer.

In contrast to these results, NAC and ebselen (a glutathione peroxidase mimic and a potent peroxide scavenger) were reported to inhibit metastasis in mouse tumor cell lines derived from Lewis lung carcinoma and in mouse fibrosarcoma B82M cells (Ishikawa et al., 2008). These cells overproduce mtROS due to several mutations in the mitochondrial genes encoding subunits of complex I of the electron transport chain. These mutations cause ROS-dependent activation of the MCL-1 gene (antiapoptotic) as well as the pro-angiogenic HIF-1a and VEGF genes associated with high metastatic potential. The reversal of the metastatic phenotype by antioxidants indicates that metastases are activated by ROS-dependent gene expression rather than by accelerating genetic instability. Systematic studies have identified hundreds of mtDNA variants potentially associated with cancer, but the cancer inducer status of these mutations remains to be established (Kopinski et al., 2021).

The Nrf2-dependent expression may also have a pro-metastatic effect. Thus, in xenograft models of human colon carcinoma HCT116 and hepatoma HuH-7, activation of Nrf2 by hypoglycemic dipeptidyl peptidase-4 (DPP-4) inhibitors (a common class of drugs used in type 2 diabetes mellitus) or by sulforaphane significantly increased the risk of metastasis without increasing the incidence or growth rate of primary tumors (Wang et al., 2016). The role of Nrf2-mediated expression of antioxidant enzymes in the pro-metastatic effect of the drugs has not been verified in the study and deserves further study.

When discussing the prometastatic effects of some antioxidants, two important points should be noted. The first one is related to the role of the lymphatic system in metastasis. It is known that many types of cancer (including melanoma) are more likely to form metastases in the lymph nodes than in distant organs. Metastases in the lymph nodes are able to release cancer cells into the bloodstream, promoting further metastasis. Importantly, cells in the lymph undergo less oxidative stress than those in the blood (Ubellacker et al., 2020). This is at least in part due to the lower concentrations of oxygen and iron in the lymph compared to the blood. As a result, the lymphatic environment protects melanoma cells from ferroptosis, a form of regulated cell death mediated by lipid peroxidation (Ubellacker et al., 2020). The low level of oxidative stress in the lymph likely modified the effects of NAC and vitamin E, resulting in increased metastasis almost exclusively to the lymph nodes.

The second point is related to the role of the immune system in carcinogenesis and metastasis. Most studies cited above were performed in xenograft models using immunocompromised NOD/SCID IL2Rγ^null^ (NSG) mice, which lack mature T cells, B cells, natural killer (NK) cells, and are deficient in multiple cytokine signaling pathways (Piskounova et al., 2015). BALB/c nude mice, which were also used, lacked T and B cells, while other defects in their immunity were not clearly identified (Wang et al., 2016). Defects in immune control of tumor growth and metastasis can significantly modify the effects of antioxidants. For example, in contrast to the results of (Wang et al., 2016), it was shown that antidiabetic DPP-4 inhibitors have a pronounced antimetastatic activity when used in wild-type animals (Jang et al., 2015). This effect is likely due to enhanced natural immunity to tumors (Barreira da Silva et al., 2015).

Antioxidants can stimulate the antimetastatic and antitumorigenic effects of cytotoxic cytokines, such as tumor necrosis factor (TNF) and TNF-associated apoptosis inducing ligand (TRAIL) by preventing development of cell resistance. There are several mechanisms of resistance, but one of the most important is associated with the activation of the transcription factor NF-kB induced by TNF and TRAIL in parallel with the initiation of apoptotic signaling (Falschlehner et al., 2007; Brenner et al., 2015). NF-kB stimulates a variety of anti-apoptotic mechanisms as well as the expression of pro-inflammatory cytokines, including TNF. As a result, NF-kB inhibitors stimulate TNF and TRAIL-induced apoptosis (Jeremias et al., 1998; Van Antwerp et al., 1998). The clinical application of inhibitors of NF-kB signaling is being intensively studied (Ramadass et al., 2020). Natural antioxidants, including flavonoids, carotenoids, and NAC, effectively inhibit NF-kB activation (Staal et al., 1990; Bremner and Heinrich, 2002). The same effect has been described for mitochondria-targeted antioxidants (Hughes et al., 2005; Zakharova et al., 2017a), indicating that mtROS production is critical for NF-kB activation.

## 4 Mitochondria-targeted antioxidants

**FIGURE 1:**
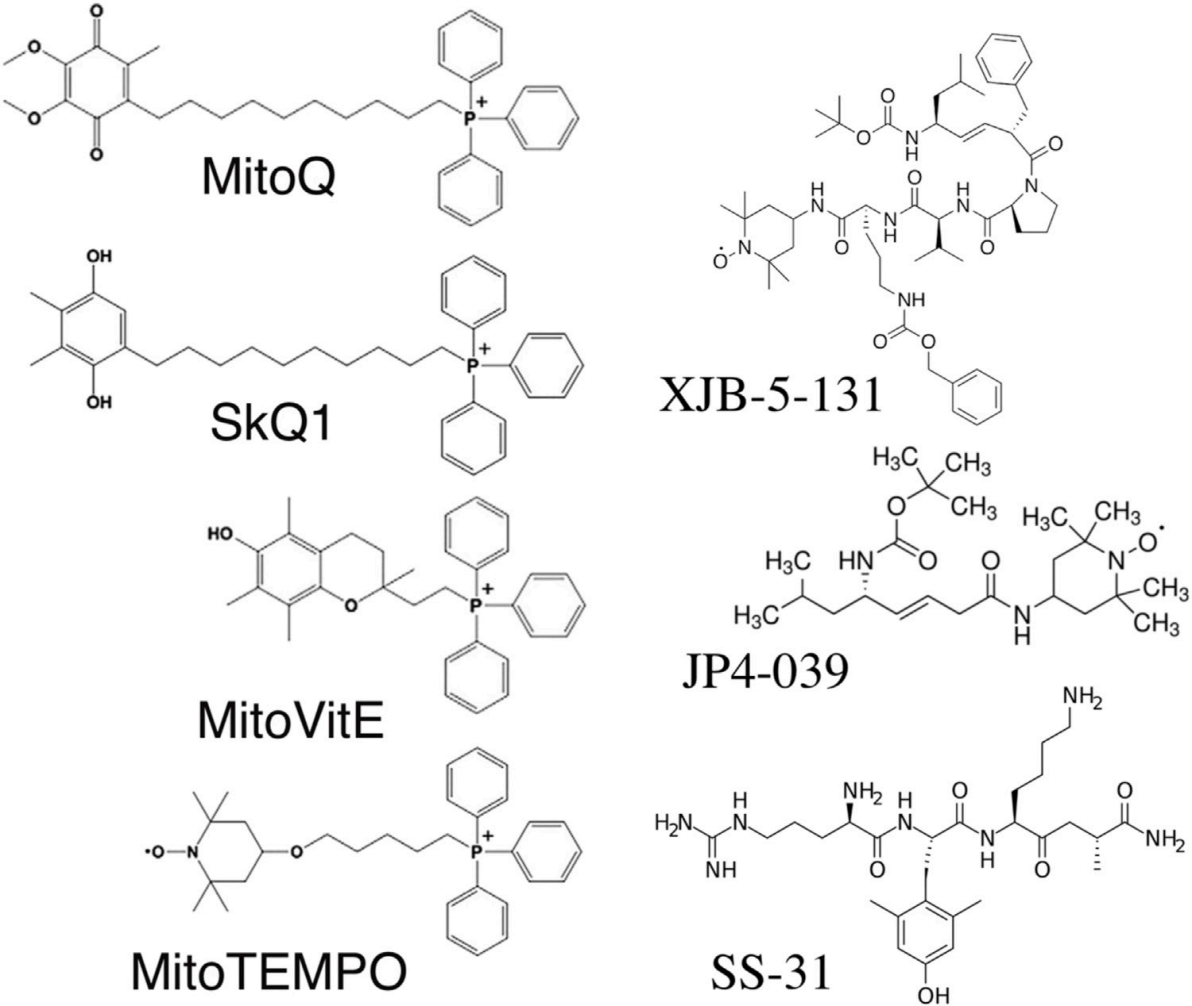
Structure of mitochondria-targeted antioxidants.

Mitochondria are the only cellular organelles with a negative charge inside. This unique property led to the discovery of mitochondria-penetrating positively charged cations (Liberman and Skulachev, 1970). These penetrating cations were able to cross cellular membranes and selectively accumulate within mitochondria. The latter fact allowed their use as “electric locomotives” to target other compounds into mitochondria (Skulachev, 1988). The most widely used penetrating cation is triphenylphosphonium linked to a long alkyl chain (Figure 1). The first mitochondria-targeted antioxidant, thiobutyltriphenylphosphonium bromide, was synthesized in 1995 by M. P. Murphy and coworkers (Burns et al., 1995). Later, the thiobutyl moiety was replaced by vitamin E (Figure 1). The resulting antioxidant very efficiently protected mitochondria from oxidative damage (Smith et al., 1999). This antioxidant irreversibly loses its antioxidant properties after a single interaction with ROS. The next milestone was the creation of the “rechargeable” antioxidant MitoQ, able to be converted back into its reduced form by mitochondrial electron transport chain (Figure 1) (Kelso et al., 2001). Afterward, the whole family of SkQ compounds (Figure 1) was synthesized, containing a highly efficient plastoquinone antioxidant, which is also readily reduced by the, ETC. MitoQ and SkQ1 are the most studied mitochondria-targeted antioxidants, demonstrating efficacy both *in vitro* and *in vivo* [reviewed in (Zinovkin and Zamyatnin, 2019)]. SkQ1 has been successfully used in preclinical studies for the treatment of cardiovascular and renal diseases (Bakeeva et al., 2008; Plotnikov et al., 2019), and has also demonstrated anti-inflammatory activity in acute bacterial infection (Plotnikov et al., 2013) and in the systemic inflammatory response syndrome (SIRS) model (Zakharova et al., 2017b). The high efficiency of eye drops containing SkQ1 has been demonstrated not only in various models of eye diseases in animals (Neroev et al., 2008), but also in a clinical study of dry eye syndrome (Brzheskiy et al., 2015). SkQ1 eye drops (Visomitin) are registered and marketed for the treatment of dry eye syndrome. To date, about a dozen mitochondria-targeted antioxidants have been synthesized, used in research, and are undergoing clinical trials (Zielonka et al., 2017).

### 4.1 SkQ1

The first study of the antitumor effects of a mitochondria-targeted antioxidant was carried out using SkQ1 (Agapova et al., 2008). It was found that 5 nmol SkQ1/kg per day (obtained with drinking water) delayed the appearance of tumors (mainly T- and B-lymphomas) in p53^−/−^ mice (C57BL/6 background). The lifespan of the animals increased by 30%–40%, in line with earlier observations that lymphomas were the main cause of death in these mice (Sablina et al., 2005). It was previously shown that homozygous p53 gene knockout in mice is accompanied by a significant increase in ROS production (Sablina et al., 2005). SkQ1 caused an approximately twofold decrease in the intracellular ROS level in p53 ^−/−^ mouse tissues, in particular, in spleen cells. It is still unclear whether HIF-1α stabilization is involved in the antitumor effect of SkQ1.

A similar study, when SkQ1 at various doses (0.5–2,500 nmol/kg day) was applied to female 129/Sv mice, both outbred and inbred, did not reveal any effect on spontaneous tumor formation with a significant increase in median lifespan (Iurova et al., 2010). SkQ1 also had no effect on the high incidence of mammary tumors in transgenic mice overexpressing HER2/neu (Iurova et al., 2010). Later, the influence of SkQ1 on the development of spontaneous tumors was tested in wild-type BALB/c mice (Manskikh et al., 2014). The BALB/c mice are characterized by an extremely high incidence of various hematopoietic tumors (lymphomas and leukemias) with a very low incidence of breast tumors, which significantly distinguishes this line from the previously studied line C57BL/6. It was shown that SkQ1 (1–30 nmol/kg per day) did not significantly affect the overall incidence of hematopoietic tumors in males and females. At the same time, SkQ1 modified the spectrum of spontaneous tumors in female mice, reducing the incidence of follicular lymphomas. SkQ1 also suppressed the spread of lymphomas, but did not significantly affect the overall incidence of benign and malignant tumors or the lifespan of mice with tumors. Importantly, both studies cited demonstrate that SkQ1 does not have carcinogenic or tumor-promoting effects.

The effect of SkQ1 on tumor growth was studied on xenografts of human colon carcinoma cells of the HCT116 line in athymic mice (Agapova et al., 2008). SkQ1 did not affect the growth of these tumors, but significantly inhibited the growth of tumors derived from p53 knockout HCT116 cells. SkQ1 also did not inhibit the increase in the volume of human cervical carcinoma xenografts (SiHa cell line), but significantly increased lifespan of mice with tumors (Agapova et al., 2008). SkQ1 (5 nmol/kg per day) was shown to be ineffective in suppressing tumor growth after orthotopic injection of syngeneic tumor cells (Panc02 cell line) into the pancreatic head of C57/BL6 mice, a model of pancreatic duct adenocarcinoma (Bazhin et al., 2016). The most pronounced effect of SkQ1 was observed on tumor growth in xenografts of human rhabdomyosarcoma (cell line RD, ATCC # CCL-136) (Titova et al., 2018). This effect was achieved at a higher dose of SkQ1 (250 nmol/kg every 2 days) with the introduction of the substance into the esophagus through a catheter. A very high sensitivity of rhabdomyosarcoma cells to SkQ1 has also been demonstrated *in vitro* (Titova et al., 2018).

Previously, in hepatocellular xenograft tumors (Gao et al., 2007) and in HCT116 cell xenografts (Khromova et al., 2009), it was assumed that antioxidants inhibit tumor growth mainly by inhibiting angiogenesis. It was shown that SkQ1 inhibits angiogenesis *in vivo* in the matrigel subcutaneous implant model (Agapova et al., 2008). The content of relatively large (>40 μm) vessels decreased significantly at 100 nmol SkQ1/kg per day, while the content of smaller vessels decreased only at doses 10–100 times higher. In HCT116/p53^−/−^ tumor xenografts, treatment with 5 nmol SkQ1/kg per day caused a significant increase in the number of underdeveloped lumen-free blood vessels, but only a slight decrease in the number of typical vessels. These data indicated that SkQ1 strongly suppressed vascular maturation, while their invasion was less sensitive. They are consistent with the possible inhibition of HIF1α by SkQ1, as shown for other mitochondrial-targeting antioxidants (Choudhry and Harris, 2018).

SkQ1 inhibited benzo(a)pyrene-induced soft tissue carcinogenesis as well (Anikin et al., 2013). SkQ1 (5–50 nmol/kg per day) reduced average tumor volume by twofold and increased mouse survival rate in this model. The genotoxic mechanism of benzo(a)pyrene includes oxidative metabolism to highly reactive compounds that form covalent adducts with DNA. Therefore, one cannot rule out the direct antioxidant effect of SkQ1 on this process. For example, dietary antioxidants curcumin and vitamin E have been shown to protect against the DNA-damaging effects of benzo(a)pyrene in lung epithelial cells (Zhu et al., 2014).

The anti-tumor mechanisms of SkQ1 were studied *in vitro* using various cell lines. SkQ1 (5–50 nM) inhibited the proliferation of HCT116/p53−/− and SiHa carcinomas containing wild-type p53 but suppressed p53-dependent signaling caused by HPV16 papillomavirus infection (Agapova et al., 2008). SkQ1 did not affect either the levels of cyclin-dependent kinase (Cdk) inhibitors p21Cip1/Waf1 and p27Kip1, or the expression of p53-responsive genes. The mechanisms of growth inhibition caused by SkQ1 have been further studied in cervical carcinomas SiHa and CaSki (Shagieva et al., 2017). SkQ1 was found to inhibit the activation of extracellular signal-regulated kinases 1 and 2 (ERK1/2), members of the mitogen-activated protein kinase (MAPK) family. These kinases are known to regulate the proliferation of various cell types. However, the same doses of SkQ1 were shown to have no effect on the proliferation of non-malignant HaCaT cells. Epidermal growth factor (EGF) stimulates ERK1/2 as well as the proliferation of carcinoma cells. Surprisingly, SkQ1 suppressed EGF-induced ERK1/2 activation in SiHa cells, but not in Ca-Ski cells. This difference is likely due to the fact that the expression of the EGF receptor in Ca-Ski cells is about 6 times higher than in SiHa cells (Bachran et al., 2010).

Probably, the SkQ1-induced inhibition of the proliferation of carcinoma cells is associated with the differentiating effect of SkQ1 (see below). It is known that some differentiation markers can regulate cell cycle progression. In carcinomas, the most important is E-cadherin/β-catenin signaling, leading to inhibition of Cdk (Pugacheva et al., 2006). In all studied carcinomas, SkQ1 induced the expression of E-cadherin and changes in the cytoskeleton, indicating the stimulation of epithelial differentiation (Agapova et al., 2008; Shagieva et al., 2017). It is important to note that SkQ1-dependent expression of E-cadherin in SiHa and CaSki was mediated by inhibition of ERK1/2 (Shagieva et al., 2017). Therefore, the two mechanisms of inhibition of proliferation may be interrelated.

Another mechanism of SkQ1-induced inhibition of proliferation has been described in fibrosarcoma and rhabdomyosarcoma cells (Titova et al., 2018). It was shown that SkQ1 (20–40 nM) strongly inhibits the growth of HT1080 fibrosarcoma and increases the population of multinucleated cells by more than 10 times. The mitotic rate in partially synchronized HT1080 cells decreased more than threefold after incubation with SkQ1. Cell cycle analysis showed that SkQ1 decreased the G1 (2n) cell population and significantly increased the polyploid cell population. Time-lapse microscopy showed that SkQ1 increases the duration of mitotic phases, in particular telophase/cytokinesis. In control HT1080 cells, a population with prolonged mitosis and delayed telophase/cytokinesis stages was also found, but SkQ1 increased this population by almost 10 times (up to 60%). This effect of SkQ1 on mitotic cells may be responsible for inhibition of proliferation. It is important to note that in normal diploid fibroblasts, SkQ1 did not affect either proliferation or modified mitosis.

The prolonged mitosis induced by SkQ1 in HT1080 cells may be mediated by its effect on the anaphase stimulating complex (APC). The activated APC regulates the transition of cells from metaphase to anaphase and is responsible for the inactivation of cyclin-dependent kinases. Finally, APC regulates kinases of the Aurora family and completes the cytokinesis process (Lindon et al., 2015). SkQ1 treatment significantly reduced the activation (phosphorylation) of all three Aurora isoforms (A, B, C) in partially synchronized cells. Moreover, SkQ1 inhibited Aurora B translocation to the furrow area.

Cytokinesis is regulated by several protein kinases, including Aurora-B and Rho-associated kinase (ROCK). The tumor phenotype of human fibrosarcoma cells HT1080 is caused by the activated N-RAS oncogene responsible for modification of Rho-dependent signaling (Paterson et al., 1987). An activated RAS increases the production of mitochondrial ROS, which is important for carcinogenesis (Weinberg et al., 2010; Liou et al., 2016). Earlier, in Ras-transformed fibroblasts (Agapova et al., 2008), as well as in HT1080 cells (Titova et al., 2018), the scavenging of mitochondrial ROS using SkQ1 induced a Rho-dependent reorganization of the actin cytoskeleton in HT1080 cells. This effect of SkQ1 probably also contributes to the delay of mitosis and incomplete cytokinesis.

One more possible mechanism of SkQ1-dependent inhibition of HCT1080 cell proliferation may be associated with the activation of the Rb retinoblastoma protein. Because Rb is involved in the regulation of cell proliferation, tumor development is frequently associated with Rb inactivation (Rubin et al., 2020). Rb can be inactivated by various mechanisms, including phosphorylation and degradation. SkQ1 significantly reduces the amount of phosphorylated Rb in synchronized HT1080 cells (Titova et al., 2018) and thus can inhibit proliferation.

In human rhabdomyosarcoma cells (RD cell line), as well as in HCT1080 cells, SkQ1 induced prolonged mitosis, suppressed cytokinesis and stimulated multinucleated cell formation (Titova et al., 2018). SkQ1 also inhibited phosphorylation of Rb in synchronized RD cells but in contrast to HCT1080 cells, SkQ1 induced apoptosis of RD cells. To our knowledge, it was the first example of pro-apoptotic action of mitochondria-targeted antioxidants. SkQ1’s effect was not mediated by pro-oxidant activity because i) effective concentrations were much lower than pro-oxidant (micromolar) concentrations, and ii) antioxidants NAC (1 mM) and Trolox (0.1 mM) also induced apoptosis in RD cells. C12TPP, an analog of SkQ1 without the antioxidant (plastoquinone) group, did not influence proliferation and apoptosis of HT1080 and RD cells. C12TPP, an analog of SkQ1 without antioxidant (plastoquinone) group, did not influence proliferation and apoptosis of HT1080 and RD cells. Uncoupler FCCP that dissipates mitochondrial membrane potential and therefore prevents accumulation of SkQ1 in mitochondria prevented SkQ1-induced apoptosis of RD cells supporting the conclusion on the critical role of mitochondrial ROS in survival of these cells (Titova et al., 2018). It seems reasonable to suggest that SkQ1-induced arrest of the cell cycle induced apoptosis of RD cells, but this possibility was not analyzed in the experiments. Another example of apoptosis stimulated by SkQ1 was reported recently for neutrophils (Vorobjeva et al., 2017). However, apoptotic mechanisms in these non-dividing cells are clearly different from cancer cells.

The carcinogenesis of epithelial tumors depends on the rearrangement of the cytoskeleton and adhesion junctions (AJ), because of which they acquire the morphofunctional characteristics of mesenchymal cells. This process, known as epithelial-mesenchymal transition (EMT), confers migratory, invasive, and stem cell properties to epithelial cells. EMT is involved in morphogenesis during embryonic development and in organ fibrosis (Thiery, 2002; Ye and Weinberg, 2015). Shagieva et al. (2017) found that SkQ1 restores some properties of the epithelium in human cervical carcinomas SiHa and Ca-Ski. These data indicate that mitochondrial ROS are important components of EMT signaling in tumors. SkQ1 (40 nM, 3 days) was shown to induce actin stress fibers and circumferential actin rings, resulting in morphological changes and the formation of epithelial islets. SkQ1 treatment reduced cell motility in Transwell assays. Importantly, SkQ1 does not alter the general organization of the actin cytoskeleton in non-tumorigenic HaCaT keratinocytes. Adhesion junctions connected to β-actin are severely disorganized in both carcinoma cell lines, and SkQ1 partially restored their structure. SkQ1 stimulated the expression of E-cadherin, the main component of AJ, and induced its redistribution in the cell-cell contact area, where it was co-localized with β-actin. In the Ca-Ski cell line, which was derived from metastatic cervical carcinoma (while SiHa cells were derived from the primary tumor), SkQ1 inhibited the expression of mesenchymal N-cadherin, a marker of advanced EMT. E-cadherin expression is suppressed by the Snail, Slug, and Twist transcription factors, which are activated during EMT (Serrano-Gomez et al., 2016). SkQ1 induced a significant decrease in Snail expression in SiHa cells, but not in Ca-Ski cells, where Snail expression was much lower. Snail can be activated by the ERK1/2 pathway (Montserrat et al., 2012), so SkQ1-dependent inhibition of ERK1/2 may be responsible for AJ restoration (Shagieva et al., 2017).

EGF receptor (EGFR) mediated signaling is known to activate EMT in cervical carcinogenesis (Lee et al., 2008). EGF stimulated ERK1/2 and Snail in SiHa and Ca-Ski cells, resulting in an advanced EMT phenotype. SkQ1 inhibits the effects of EGF in SiHa cells, but not in Ca-Ski cells (Shagieva et al., 2017), probably because the expression of EGFR in Ca-Ski cells is higher than in SiHa cells (Bachran et al., 2010). It is likely that the pathway that regulates the expression of the transcription factor Twist1, which regulates EMT in distant metastases (Lee and Nelson, 2012), may be a target for SkQ1 in Ca-Ski cells, but this possibility requires further study.

The above data on the role of mitochondrial ROS in EMT were independently confirmed in a study in which overexpression of mitochondria-targeted catalase in transgenic mice with metastatic breast cancer reduced primary tumor invasiveness significantly (Goh et al., 2011). In aggressive breast cancer cell lines, knockdown of the mitochondrial superoxide dismutase (MnSOD/SOD2) gene has been shown to reverse EMT, indicating that hydrogen peroxide produced by MnSOD in mitochondria promotes EMT (Loo et al., 2016).

As mentioned above, initial rearrangement of the actin cytoskeleton during EMT is controlled by small GTPases of the Rho family. Mitochondrial ROS can modulate Rho-dependent changes in the actin cytoskeleton: inhibition of mtROS by SkQ1 restored actin stress fibers in Ras-transformed fibroblasts (Agapova et al., 2008); another mitochondria-targeted antioxidant, MitoTEMPO, prevented RhoA/ROCK activation in endothelial cells (Chu et al., 2017). Another important target of SkQ1 was identified in the study of myofibroblast differentiation (Popova et al., 2010). This differentiation resembles the extreme case of EMT and depends on transforming growth factor 1 (TGF*b*1), which is also one of the key EMT factors (Docherty et al., 2006). Exogenous TGF*b*1 induced the formation of myofibroblasts in the culture of subcutaneous fibroblasts, while SkQ1 prevented this effect (Popova et al., 2010). TGFb1-dependent and NFkB signaling are implicated in the activation of matrix metalloproteinases (MMPs), which, together with EMT, are essential for cancer cell invasion and metastasis (Moore-Smith et al., 2017). In endothelial cells, SkQ1 has been shown to inhibit NF*k*B activation (Zinovkin et al., 2014) and NFkB-dependent MMP9 activation (Zakharova et al., 2017a). Inhibition of EMT and MMP activation by mitochondria-targeted antioxidants can be considered a promising strategy for suppressing metastasis.

Using a fluorescent analogue of SkQ1 carrying the cationic group of rhodamine-19 (SkQR1), it was shown that P-glycoprotein (Pgp170) effectively extrudes SkQR1 molecules from cells (Agapova et al., 2008; Fetisova et al., 2010). As a result, the effectiveness of SkQR1 and SkQ1 as protective agents against hydrogen peroxide toxicity was strongly suppressed in cells expressing Pgp170. This pump extrudes a wide range of hydrophobic, positively charged molecules from cells; therefore, this property of SkQR1 and SkQ1 is probably common to many other mitochondria-targeted antioxidants. It has been suggested that the extrusion of antioxidants from tumor cells can be used for the therapy of multidrug-resistant (MDR) tumors. This is possible if antioxidants protect normal cells (with low Pgp170 levels) from destruction by X-rays, photodynamic therapy, or chemotherapy mediated by severe oxidative stress, while the destruction of tumor cells (with high Pgp170 levels) is not affected. This possibility was illustrated *in vitro* using SkQR1 as a radioprotective agent (Fetisova et al., 2015). It was shown that SkQR1 (20 nM) prevents the accumulation of DNA double-strand breaks and chromosomal aberrations in erythroleukemia K562 cells exposed to gamma irradiation (1.5 Gy). In subline K562 with overexpression of Pgp170, the protective effect of SkQR1 was not found. The selective protection of MDR-negative normal cells could probably increase the radiation doses to tumors and increase the effectiveness of radiation therapy.

### 4.2 MitoQ

The proliferation of two breast cancer cell lines, MDA-MB-231 and MCF-7, was inhibited by MitoQ, the pioneering mitochondria-targeted antioxidant, with IC50 values of 296 nM and 113 nM, respectively. At the same time, MitoQ at concentrations up to 10 μM had no effect on the proliferation rate of the non-tumor MCF12A cell line. These findings were confirmed by colony formation assays, but the effective concentrations of MitoQ were 5–10 times higher (Rao et al., 2010). However, these promising results were attributed to the prooxidant rather than antioxidant activity of MitoQ. MitoQ has been shown to stimulate ROS production in breast cancer cells, and these ROS modify thiols in Keap1, activating Nrf2-dependent transcriptional activity (Rao et al., 2010). The same mechanism of toxicity caused by MitoQ (2 μM) was described for MDA-MB-231 and the H23 lung cancer cell line (Pokrzywinski et al., 2016). This study found excessive production of mitochondrial ROS, damage to mitochondrial DNA, and inhibition of mitochondrial respiration. The prooxidant and inhibitory effects of MitoQ underly the inhibition of the growth of human melanoma cell line B-Raf^V600E^ with IC50 values of 300–700 nM (Hong et al., 2017). The predominant prooxidant effect of MitoQ in these models probably reflects a very narrow (compared to SkQ1) window between the concentrations that induce anti- and prooxidant effects both in cell-free systems and in various cells (Antonenko et al., 2008).

The antioxidant properties of MitoQ have been reported to suppress neoplastic transformation in a model of acinar-to-ductal metaplasia induced by active Kras expression (Liou et al., 2016). In primary acinar cells infected with the lentiviral oncogenic Kras, MitoQ (500 nM) prevented ROS overproduction, ROS-dependent activation of the NF-kB transcription factor, and EGFR expression. *In vivo*, MitoQ reduced KrasG12D-induced formation of abnormal pancreatic structures as well as EGFR expression. The effect was observed after intraperitoneal administration of MitoQ adsorbed on cyclodextrin to mice at a dose of 20 mg/kg every 2nd day for 12 weeks. The antioxidant effect of MitoQ *in vivo* was confirmed by a decrease in Nrf2 expression. The predominant antioxidant activity of MitoQ in this model is probably associated with strong stimulation of mitochondrial ROS production by Kras.

### 4.3 MitoVitE

Alpha-tocopherol (vitamin E) was first conjugated with triphenylphosphonium in 1999 (Smith et al., 1999), but the anticancer activity of this compound (MitoE, MitoE2, MitoVitE) has not been tested until recently. In a recent study, MitoVitE (100 nM) was found to inhibit the growth of human T Cell acute lymphoblastic leukemia cell line Jurkat when complex I (but not complex III) of the mitochondrial respiratory chain was inhibited by a specific inhibitor, piericidin (Kong et al., 2020). It has been shown that inhibition of Complex I in combination with MitoVitE suppresses the formation of mitochondrial ROS, induces strong metabolic changes and an integrated stress response, including the cytoplasmic (but not mitochondrial) unfolded protein response. Further research is needed to decipher the signaling pathways modulated by MitoVitE in this model.

The cytotoxicity of a mitochondria-targeted vitamin E analogue with an extended linker between tocopheryl and TPP^+^ moieties (called mitochromanol, Mito-ChM) has been tested in various breast cancer cell lines (Cheng et al., 2013). It was shown that Mito-ChM inhibits the growth of these cells with maximum efficiency (IC50 4.9 μM) for the MDA-MB-231 cell line and is ineffective in the non-tumorigenic MCF10A cell line up to 20 μM. The prooxidant effect of Mito-ChM (2 μM) was confirmed in cell lines MDA-MB-231 and H23, where excessive mitochondrial ROS production, damage to mitochondrial DNA, and inhibition of mitochondrial respiration were observed (Pokrzywinski et al., 2016). In a xenograft model with MDA-MB-231 cells, after treatment with Mito-ChM (60 mg/kg five times a week for 4 weeks), tumor mass decreased by 30% compared to control mice (Cheng et al., 2013). As in the case of MitoQ, the cytotoxic effect of Mito-ChM was associated with a prooxidant and inhibitory effect, since its acetylated ester analog, devoid of antioxidant activity, has the same toxicity towards breast cancer cells. Another esterified (succinylated) mitochondria-targeted analogue of vitamin E (MitoVES) lacking antioxidant activity also demonstrates a cytotoxic effect both *in vitro* and in a xenograft model with HCT116 colon carcinoma cells. This effect was mediated by specific inhibition of mitochondrial complex II (Dong et al., 2011).

### 4.4 MitoTEMPO

The first mitochondria-targeted nitroxide MitoTEMPO (2,2,6,6-tetramethyl-4- [5- (triphenylphosphonio) pentoxy] piperidine-1-oxybromide) was synthesized in 2008 (Trnka et al., 2008), but its slightly modified analog MitoTEMPO (2- (2,2,6,6-tetramethylpiperidin-1-oxyl-4-ylamino) -2-oxoethyl) triphenylphosphonium chloride) is becoming more and more popular in further research. It has been suggested that the antioxidant effects of these nitroxides may be mediated by their reduction to hydroxylamines by ubiquinol in mitochondria (Trnka et al., 2008). It was also shown that MitoTEMPO has SOD-like activity and acts as a mitochondria-targeted SOD mimetic (Dikalova et al., 2010).

It was shown that MitoTEMPO (5–50 nM) inhibits cell growth and induces apoptosis in B16-F0 mouse melanoma cells (Nazarewicz et al., 2013). For some unknown reason, the maximum effect was observed with 25 nM MitoTEMPO. MitoTEMPO at this concentration reduced the activation of Akt and Erk 1/2, which was measured by their phosphorylation. It has been suggested that the antiproliferative and proapoptotic actions of MitoTEMPO may be based on inhibition of kinase-dependent pathways. In addition, MitoTEMPO has been shown to inhibit glycolysis and reduce the level of ATP in melanoma cells. In a xenograft model, MitoTEMPO (1.5 mg/kg/day), administered using implanted osmotic pumps, inhibits the growth of established tumors induced by the human melanoma cell line A375 (Nazarewicz et al., 2013).

MitoTEMPO in low doses suppresses nitrosodiethylamine-induced hepatocarcinogenesis in mice (Shetty et al., 2019). Intraperitoneal administration of mitoTEMPO (0.1 mg/kg once every week) 2 weeks prior to carcinogen and then for 20 weeks, increased the survival rate of animals by 30%, reduced the incidence of tumors (25%) and the multiplicity of tumors (39%) compared with control mice. MitoTEMPO significantly increased the level of reduced glutathione and inhibited lipid peroxidation in the livers of mice treated with nitrosodiethylamine, indicating that the suppression of carcinogenesis was mediated by the antioxidant effect of mitoTEMPO.

Later, cytotoxicity of MitoTEMPO at very high concentrations (IC50 100 μM) was shown in the lung adenocarcinoma cell line A549 (Dias Amoedo et al., 2020). Proteomic analysis of cells treated with 10 μM MitoTEMPO revealed some signs of antioxidant action as well as changes in the expression of several cancer-related genes. In the model of super invasive SiHa and B16 cells (selected *in vitro* or *in vivo*, respectively), MitoTEMPO suppressed cell migration at a high (50 μM) concentration, but the role of its antioxidant activity remained unclear (Porporato et al., 2014). In the same study, pretreatment of B16 cells with 50 μM MitoTEMPO inhibited lung metastases in syngeneic mice 15 days after cell injection. In addition, the authors reported that the number of lung metastases observed 30 days after implantation of the primary tumor was significantly reduced in mice treated with MitoTEMPO (0.7 mg/kg per day, intraperitoneal injections).

Interestingly, very high concentrations of mitoTEMPO (200–400 μM) have been shown to produce the same effect as MitoVitE (100 nM) in Jurkat cell line when complex I is inhibited (Kong et al., 2020). Even at these extremely high concentrations, MitoTEMPO reduces mitochondrial ROS production in the presence of piericidin. In addition, MitoTEMPO (5 mg/kg per day) in combination with the Complex I inhibitor phenformin (75 mg/kg per day) inhibited the accumulation of T Cell acute lymphoblastic leukemia (Jurkat and MOLT-4 cell lines) in the spleen, bone brain, and peripheral lymph nodes compared to control mice. Both MitoTEMPO and phenformin were ineffective when given separately. A high concentration of MitoTEMPO (100 μM) has been shown to inhibit anchorage-independent growth of MCF7 breast cancer cell line overexpressing c-Myc and H-Ras (G12V) (Ozsvari et al., 2017). However, the same group later reported that non-targeted 4-hydroxy-TEMPO (100 μM) had the same effect (De Francesco et al., 2017).

Brand and colleagues used chemical library screening to find a new class of compounds that suppress mitochondrial ROS generation at various locations of the respiratory chain without altering respiration or oxidative phosphorylation (Brand et al., 2016). Suppressors of ROS production in the I_Q_ site (upstream of the quinone-binding rotenone-sensitive site) in complex I (S1QEL) suppress ROS production in various cell models and protect against ischemia-reperfusion injury in the perfused mouse heart (Brand et al., 2016). One of these compounds [5- (4-methoxyphenyl) -3H-1,2-dithiol-3-thione] was tested *in vitro* and *in vivo* lung cancer models in parallel with MitoTEMPO (Dias Amoedo et al., 2020). S1QEL cytotoxicity was demonstrated on human lung adenocarcinoma cells A549, where the effect reached 40%. In seven (out of eight tested) additional human lung cancer cell lines, the effect was confirmed, but did not exceed 30%. The anti-cancer properties of S1QEL were then confirmed in the model of anchorage-independent growth of A549 cells. S1QEL also inhibited the migration of these cells in the Transwell assay. Expression of cancer-related genes was modulated by S1QEL, and these changes partially overlapped with the effect of MitoTEMPO. Finally, in an orthotopic mouse xenograft model with A549 human lung adenocarcinoma cells, a significant (almost 2-fold) reduction in tumor size was observed in animals treated with S1QEL (10 mg/kg, daily intraperitoneal injections) compared to control mice. More research is required to fully understand the antitumor effect of S1QEL, including the potential suppression of metastasis.

### 4.5 Peptide-based mitochondria-targeted antioxidants

Some antioxidants have been targeted to mitochondria using peptides that have a high affinity for the mitochondrial membrane. 4-amino-TEMPO was conjugated with fragments of the cyclopeptide antibiotic gramicidin S (Fink et al., 2007; Rajagopalan et al., 2009). These molecules (XJB-5-131, JP4-039) accumulate in the mitochondria of cells and protect them against prooxidants and gamma radiation *in vitro* and *in vivo* (Rajagopalan et al., 2009; Goff et al., 2013).

The series of peptides known as Szeto-Schiller (SS) peptides have been shown to accumulate in mitochondria and exhibit some antioxidant activity (Szeto and Schiller, 2011). Initially, it was assumed that this effect is mediated by ROS scavenging activity of the dimethyltyrosine (Dmt) residue, but later it was shown that the effect persists after replacing Dmt with phenylalanine lacking scavenging activity (Yang et al., 2009). It was suggested that the effects of SS peptides are due to the interaction with cardiolipin (CL) and the destruction of the CL-cytochrome *c* complex (Szeto, 2014). It was previously established that this complex has peroxidase activity and may be responsible for mitochondrial lipid peroxidation (Kagan et al., 2005).

To our knowledge, peptide-based mitochondria-targeted antioxidants have never been tested as anticancer agents. However, due to their pronounced radioprotective activity, they can be considered as a component of radiation therapy for tumors based on the principles described above for SkQ1 and SkQR1. It has been reported that oral administration of JP4-039 results in radioprotection of the oral cavity in radiosensitive Fanconi Anemia (FA) Fancd2 ^−/−^ mice with orally established TC-1 epithelial cell tumors (Shinde et al., 2016). No radioprotection of tumors with JP4-039 was observed. More recently, the radioprotection of the oral cavity and bone marrow using JP4-039 has been described for other FA genotypes, including the most common human genotype Fanca^−/−^ (Willis et al., 2018).

In mice with C26 colon cancer treated with oxaliplatin and 5-fluorouracil, SS-peptide (SS-31) restored intracellular ATP levels, reduced muscular atrophy, and metabolic alteration (Ballaro et al., 2021). SS-31 also protects against doxorubicin (DOX) -induced cardiotoxicity (Zhang et al., 2021), but the possible interference with the chemotherapeutic effect of DOX has not been analyzed.

## 5 Conclusion

Early steps of cancer initiation are accompanied by increased ROS production, and proper use of antioxidants may inhibit this process. Cell proliferation, migration, invasion, and metastasis are also dependent on ROS, indicating promise for antioxidant-based therapies. At the same time, ROS are involved in anoikis, which is an important mechanism for preventing metastasis, so antioxidants can in some cases promote metastasis. There is still no consensus on the use of conventional antioxidants, as recent results from clinical trials of Vitamin E and selenium have not shown a reduction in cancer incidence. It is not clear to what extent selenium and vitamin E actually suppressed oxidative stress in patients. At the same time, there are many examples of the anticancer activities of Nrf2-activating compounds, which reduce the oxidative load by enhancing expression of many antioxidant enzymes. Mitochondria are an important source of ROS production in cancer as well as in non-transformed cells. One of the most studied mitochondria-targeted antioxidants, SkQ1, delayed tumor appearance in p53 knockout mice and suppressed carcinogen-induced soft tissue carcinogenesis. The anticancer effects of SkQ1 may rely on the inhibition of ERK1/2 activation or the delay in mitosis and cytokinesis. Scavenging of mtROS by SkQ1 reverses EMT, which underlies the invasiveness of cancer cells. There are some other examples of successful anticancer therapy by mitochondria-targeted antioxidants in animal models indicating that targeting excessive mitochondrial ROS production is a promising strategy for cancer treatment.
